# Emerging many-body effects in semiconductor artificial graphene with low disorder

**DOI:** 10.1038/s41467-018-05775-4

**Published:** 2018-08-17

**Authors:** Lingjie Du, Sheng Wang, Diego Scarabelli, Loren N. Pfeiffer, Ken W. West, Saeed Fallahi, Geoff C. Gardner, Michael J. Manfra, Vittorio Pellegrini, Shalom J. Wind, Aron Pinczuk

**Affiliations:** 1 Department of Applied Physics and Applied Mathematics, Columbia University, New York, NY USA; 2 Department of Electrical Engineering, Princeton University, New York, NJ USA; 3 Department of Physics and Astronomy, and School of Materials Engineering, and School of Electrical and Computer Engineering, Purdue University, New York, IN USA; 4 Istituto Italiano di Tecnologia, Graphene Labs, Via Morego 30, I-16163 Genova, Italy; 5 Department of Physics, Columbia University, New York, NY USA

## Abstract

The interplay between electron–electron interactions and the honeycomb topology is expected to produce exotic quantum phenomena and find applications in advanced devices. Semiconductor-based artificial graphene (AG) is an ideal system for these studies that combines high-mobility electron gases with AG topology. However, to date, low-disorder conditions that reveal the interplay of electron–electron interaction with AG symmetry have not been achieved. Here, we report the creation of low-disorder AG that preserves the near-perfection of the pristine electron layer by fabricating small period triangular antidot lattices on high-quality quantum wells. Resonant inelastic light scattering spectra show collective spin-exciton modes at the M-point's nearly flatband saddle-point singularity in the density of states. The observed Coulomb exchange interaction energies are comparable to the gap of Dirac bands at the M-point, demonstrating interplay between quasiparticle interactions and the AG potential. The saddle-point exciton energies are in the terahertz range, making low-disorder AG suitable for contemporary optoelectronic applications.

## Abstract (web summary)

Artificial nanostructures designed to simulate models of materials such as graphene provide insights into the material physics but can also have practical advantages. Du et al. create low-disorder artificial graphene devices, and present evidence of terahertz spin-exciton modes and large Coulomb interactions.

## Introduction

Many-body effects play important roles in low-dimensional electron systems and attract great deal of attention in graphene physics due to the interplay with the honeycomb topology^1–8^. While theoretical treatments of interaction effects in graphene frequently assume that the systems are clean and controllable, these preconditions are difficult to satisfy in the natural material^1–3^. For instance, the theoretical prediction of chiral superconductivity requires tuning the Fermi energy to the M saddle-points with flatband characteristic^2^, which is difficult in graphene given the large energy gap (~5 eV, a consequence of the small lattice spacing) at the Brillouin-zone (BZ) M-point. Two-dimensional (2D) saddle-point excitons^4–7^ in graphene, redshifted from the saddle-point singularity at the M-point, are subject to prominent many-body effects, but their optical responses are at a large energy of 4.6 eV, far from the relevant energy ranges for device applications.

Artificial graphene (AG)^9–14^ is a controllable platform for simulation of quantum behavior in the physics of 2D crystals^15–19^. Linearly dispersing Dirac bands have been reported in several AG systems, including molecular assemblies on copper^11^, fermionic atoms trapped in optical lattices^12^, photonic systems^13^, and nanopatterned GaAs quantum wells (QWs)^14^. AG systems with tunable honeycomb lattices should be suitable for explorations of quantum regimes of many-body effects in graphene-like band structures. Thus far, however, electron–electron interactions in solid-state AG have not been reported, and, particularly in the case of GaAs-based AG, the low-disorder conditions for observing such effects are difficult to achieve.

Here, we report the realization of low-disorder semiconductor AG on a high-mobility GaAs QW. We fabricate small period triangular antidot lattices that significantly suppress the impact of processing disorder on electrons, and thus preserve the high-quality of states in as-grown QWs. The achievement enables observations of collective saddle-point spin excitons that are subject to exchange Coulomb interactions. For AG lattice periods in the range of tens nanometers the energy of the observed saddle-point excitons is in a much sought after terahertz range. Relatively large exchange Coulomb interactions, which have energies comparable to the gap of Dirac bands at the M-point of the BZ, could be modulated by tuning the carrier density. The capability of observing collective saddle-point spin excitons and the emergence of relatively large Coulomb interactions in the low-disorder AG lattices demonstrate access to a regime in AG that is dominated by electron–electron interaction effects, allowing the exploration of intriguing many-body effects that are inaccessible in graphene. Combining Coulomb interactions in a clean electron environment with a meV-energy gap at the M-point provides opportunities for experimental studies of many-body effects in honeycomb lattices such as chiral superconductivity^2^.

## Results

### Triangular antidot lattice

**Fig. 1:**
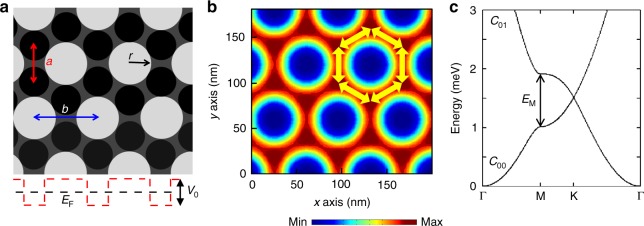
Low-disorder artificial graphene in triangular antidot lattices. **a** Schematic of a triangular antidot lattice with period *b* that is imprinted on a GaAs QW. The white circles represent etched antidots of radius *r*. The black and gray areas represent the unetched areas, and the black circles indicate the effective dots arranged in a honeycomb pattern of period *a* = *b* /$\sqrt 3$. In the evaluation of AG band structure, we used a muffin–tin potential (red dashed line) with Fermi energy *E*_F_ in the single-particle approximation^9,10,14^. *V*_0_ is the potential depth in the etched area. Each antidot produces an effectively repulsive potential *V*_0_. **b** Evaluations of electron density in a triangular antidot lattice with *V*_0_ = 6 meV, *b* = 70 nm, *a* = 40.4 nm, and *r* = 20 nm. Yellow arrows highlight strengthened electron coupling between nearest-neighbor dots. The color bar indicates the electron density. **c** The two lowest Dirac bands of AG with the parameters in **b**. *E*_M_ indicates the value of the gap near the M-point

We employ state-of-the-art nanofabrication to realize small period triangular antidot lattices in high-mobility GaAs QWs. The antidots are created by deep etching of circular holes in the semiconductor in a carefully controlled triangular lattice pattern with period *b*. Figure 1a shows that the triangular antidot lattice supports a honeycomb lattice of dots (the darker areas in Fig. 1a) with period *a*. The main advantage of this approach is twofold. First, the effective lattice period is smaller than the patterned period by a factor of $\sqrt 3$ (*a* = *b*/$\sqrt 3$). Second, electrons in the antidot lattice avoid the areas under the etched features due to a large repulsive potential, resulting in a greatly reduced impact of process-induced disorder. This is confirmed in the evaluation of electron density reported in Fig. 1b, which is a characteristic feature of the antidot triangular pattern that results in a 2D electron gas with physically connected nearest-neighbor honeycomb lattice sites in the unetched areas of the pattern (Fig. 1a, b). Figure 1c displays the calculated dispersion of the two lowest Dirac bands of AG for a typical triangular antidot lattice imprinted on a GaAs QW. The Dirac bands shown in Fig. 1c are gapless with linear momentum dispersion near the K (K’) points of the BZ and with a gap (*E*_M_) at the M-point that is comparable to the Fermi energy (~1 meV), typical of semiconductor AG.

### Device fabrication

**Fig. 2:**
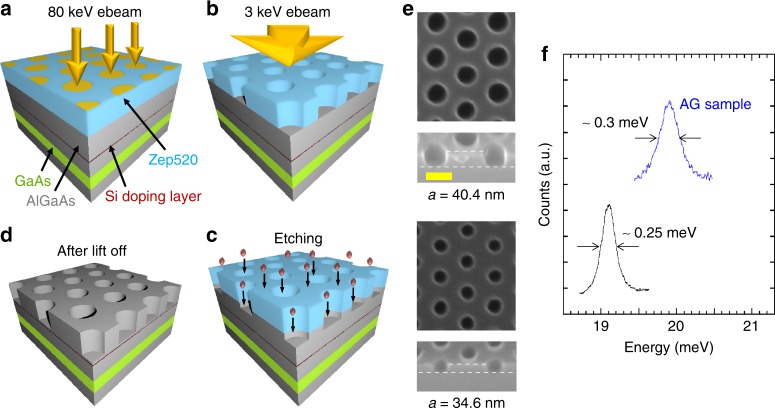
Creation of artificial graphene in triangular antidot lattices. Processing steps: **a** a Zep 520 resist is exposed by an electron beam at 80 kV accelerating voltage. After exposure, the development of the resist produces triangular antidots pattern. **b** The resist with pattern is exposed by an electron flood at 3 kV accelerating voltage, to enhance the chemical stability of the resist. **c** The BCl_3_-based dry etch is performed to transfer the pattern to the substrate with depth control. The final device after the removal of the residue resist is shown in **d**. The QW is positioned 80 nm below the surface and 30 nm below the Si δ-doping layer. The as-grown electron density is 2.1 × 10^11^ cm^−2^, with a Fermi energy *E*_F_ of 7.5 meV and a typical low-temperature mobility of 10^6^ cm^2^ V^−1^ s^−1^. **e** Scanning electron microscopy micrographs of AG lattices with different periods in 0° tilt (upper panel, top view) and 40° tilt (lower panel, side view). The white dashed lines define the etching depth. The yellow scale bar is 50 nm for all panels in **e**. **f** The comparison between the RILS spectrum of intersubband excitations of AG sample I (with incident photon energy of 1554.36 meV, blue trace) and intersubband excitations of the as-grown GaAs QW (with incident photon energy of 1550.92 meV, black trace). The peak width of intersubband excitations of sample I is nearly constant for different incident photon energies (see Supplementary Note 5 and Supplementary Figure 8). The spectra were taken at 5 K under crossed polarization

The nanofabrication of small period (60–70 nm) triangular antidot lattices, with periods much smaller than in previous works (200 nm)^20^, requires precise control of the lattice period, the antidot radius and the deep etching profile. Figure 2a–d describes schematically the processing steps involved in the creation of the antidot lattices (see Methods). Electron-beam lithography is employed to define the antidot pattern using a two-step pattern transfer process, that is, based on a Zep 520 electron-beam resist, which possesses both high-resolution capability and good resistance to etching. The e-beam writer is operated at an accelerating voltage of 80 kV to mitigate proximity effects that are relatively strong in GaAs^21–23^. A temperature-controlled, modified developing process with ultrasonication is applied (see Methods), followed by a low voltage (~3 kV) blanket electron-beam exposure to harden the resist^24^. This is followed by a BCl_3_-based inductively coupled plasma reactive-ion etching step designed to transfer the triangular antidot array to the QW. Arrays with lattice constants *b* as small as 60 nm are fabricated over an area of 200 × 200 µm^2^. Figure 2e shows top and side views of samples having patterns with *b* = 60 nm (*a* = 34.6 nm) and *b* = 70 nm (*a* = 40.4 nm) (sample parameters are listed in Supplementary Table 1). The structures are characterized by photoluminescence to determine the Fermi energy (Supplementary Figure 1). Photoluminescence outside the AG pattern where no mask is present shows that the etching depth in the sample with *a* = 40.4 nm (sample I) leads to a full depletion of electrons, suggesting that electron wavefunctions are strongly suppressed under the antidots. The estimated potential barrier is *V*_0_ = 6 meV (see Supplementary Note 1).

### Low-disorder AG

The impact of disorder resulting from nanofabrication processing is assessed by resonant inelastic light scattering (RILS)^14,19^ spectra of intersubband collective excitations^25^, in which the only electron degree of freedom that changes is the confinement state in the QW. In Fig. 2f, a comparison between intersubband excitations of sample I and the as-grown QW under crossed incident and scattered photon polarizations shows that the peak in AG has a full width at half maximum (FWHM) of 0.3 meV, which is extremely close to the FWHM ~0.25 meV of the mode in the as-grown QW (the FWHM reflects the degree of disorder in the device, which is typically due to ionized defects that scatter electrons^26^). The slight increase of FWHM could be attributed to the reduced screening of potential of ionized defects due to the greatly reduced Fermi energy (reduced charge density) in the AG sample (see Supplementary Table 1 and Supplementary Note 1). The nearly identical width of the modes in AG to that in the as-grown QW indicates the exceptional low-disorder environment for charge carriers in this small period triangular antidot lattice. This is in contrast with results from a honeycomb dot lattice with optimized fabrication processing and similar period under crossed polarization, where the FWHM increases by a factor of three from the as-grown QW value^25^.

### Low-energy Dirac-band transitions

**Fig. 3:**
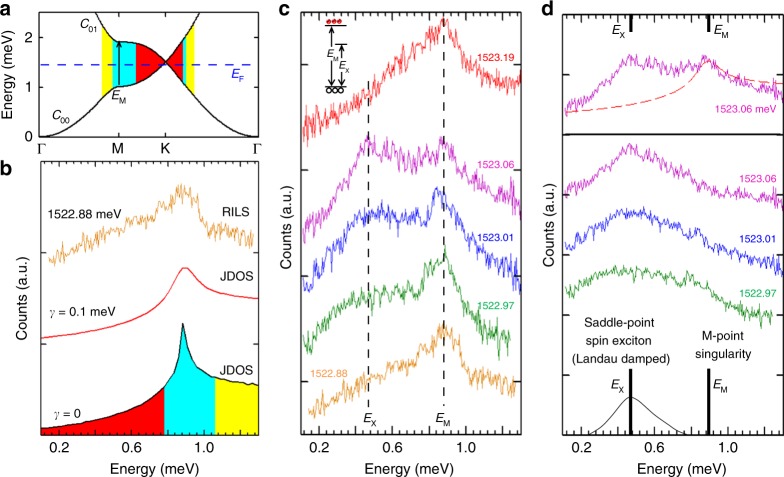
Dirac-band excitations in low-disorder AG in a triangular antidot structure. **a** AG energy band calculation with parameters in Supplementary Table 1 and diagram of Dirac-band transitions for sample I. Transitions in different regimes are illustrated by different colors. **b** The lowest plot is the joint density of states (JDOS) for the transitions in **a**. The colored areas show transitions in different regimes. The red trace is the JDOS under Gaussian broadening with width *γ* = 0.1 meV (this *γ* is chosen to fit the spectra line shape near *E*_M_). The top yellow trace is the RILS spectrum of Dirac-band excitations in sample I obtained with incident photon energy *ħω*_i_ of 1522.88 meV. **c** RILS spectra of Dirac-band excitations. A non-RILS background has been removed (subtraction details are reported in Supplementary Figures 4–6, and in Supplementary Note 3). *ħω*_i_ in meV are indicated. The vertical dashed lines indicate the positions of *E*_M_ and *E*_X._ The inset shows a level diagram for *E*_M_ and *E*_X_. In the inset, black circles are for holes in *c*_00_ band and red circles are for electrons in *c*_01_ band. **d** Top shows a RILS spectrum (purple trace) with *ħω*_i_ of 1523.06 meV, and the JDOS (red dashed line, γ = 0.1 meV) that is proportional to scattering intensity due to the single-particle transitions. Bottom: upper traces reveal the spectrum after subtraction of the single-particle JDOS intensities with *ħω*_i_ of 1523.06 meV (purple), 1523.01 meV (blue), and 1522.97 meV (green). A schematic plot shows the spin-exciton energy and the gap energy at the M-point. Due to Landau damping, the width of the spin-exciton mode increases

The AG band structure in sample I is probed by RILS measurements of low-energy excitations from Dirac-band transitions shown in Fig. 3a. RILS spectra under crossed polarization such as those in Fig. 3b, c access excitations in the spin degree of freedom^27^. Spin-density excitations involving changes in the spin degrees of freedom are redshifted from single-particle transitions due to exchange Coulomb interactions. This is an excitonic shift due to Coulomb coupling between the excited electrons and the holes in the lower state^28,29^. Charge density excitations that preserve spin orientation are active in parallel polarization where the laser tail becomes strong and obscures RILS signals in low-energy excitations. Spectra of low-energy excitations presented here are under cross polarization.

In the absence of many-body interaction effects the RILS spectra are proportional to the joint density of states (JDOS) for the transitions^14^. The two lower curves in Fig. 3b are the calculated JDOS from the parameters in Fig. 3a for sample I with *γ* = 0 meV and *γ* = 0.1 meV (*γ* is Gaussian broadening width). The evaluations show that the JDOS has a maximum at *E*_M_ = 0.9 meV (0.2 THz) arising from the saddle-point singularity for transitions at the M-point of the BZ (cyan areas in Fig. 3a, b). The rapid collapse of the RILS intensity at energies below *E*_M_, seen in the top curve in Fig. 3b, is a signature of the formation of linearly dispersing Dirac cones^14^, thus confirming the presence of a Dirac-band structure in the triangular antidot lattice in sample I.

The RILS spectrum in Fig. 3b also uncovers departures from the calculated single-particle JDOS at energies just below *E*_M_. The resonance behavior of RILS spectra shown in Fig. 3c reveals that the spectral lineshapes of low-energy Dirac-band excitations have a strong dependence on incident photon energy *ħω*_i_ (see Supplementary Note 7). Major departures of the RILS spectral lineshapes from the JDOS predictions for energies below *E*_M_ can be seen in spectra in Fig. 3d, where the single-particle JDOS has been subtracted from the spectra in Fig. 3c, revealing a relatively broad intensity maximum at *E*_X_. The redshift from *E*_M_ is interpreted as arising from many-body electron interactions that emerge in the low-disorder AG. For spin excitations in Fig. 3 the relevant many-body effects emanate from exchange Coulomb interactions. Then, it is assigned to collective spin excitations associated with the saddle-point singularity for Dirac-band transitions. An alternative interpretation for the *E*_X_ mode is discussed in Supplementary Note 6 and Supplementary Figure 9. The energy of saddle-point excitons *E*_X_ associated with transitions at the M-point singularity would be at an energy of about 0.45 meV (0.1 THz), and tunable (e.g., by changing the lattice constant).

The significant broadening of the *E*_X_ maximum (in the spectra of Fig. 3d) is due to overlap with the continuum of single-particle excitations, which causes the decay of the spin-exciton mode into electron–hole pairs (Landau damping^29^, see Fig. 3d); this is also observed at the saddle-point exciton in natural graphene^5–7^. An exchange Coulomb energy in the saddle-point exciton here can be estimated as in the case of intersubband excitations:^29^ $(E_M^2 - E_X^2)/E_{\mathrm{M}}$ = 0.6 meV. This is a relatively large exchange interaction strength that is about half of the M-point gap *E*_M_. As shown in ref. ^29^, exchange Coulomb energies are equal to *E*_ex_ = 2*nβ*, where *n* is the carrier density and β is the exchange interaction matrix element for the transition. Here, the carrier density in the *c*_00_ band is linked to the exchange energy in low-energy excitations (*c*_00_ → *c*_01_). Exchange energies in the same range could be expected in other transitions associated to the carrier density in the *c*_00_ band.

### Spin-density intersubband excitations

**Fig. 4:**
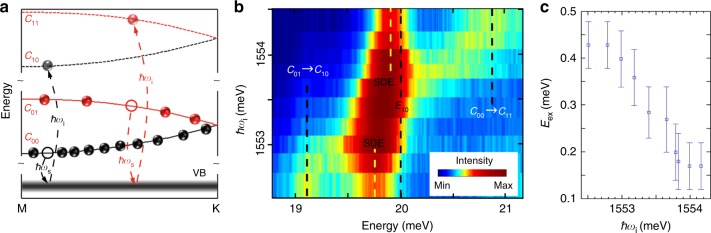
Coulomb exchange energy probed in intersubband excitations. **a** Optical transitions in RILS of intersubband spin-density excitations (SDE) are indicated. In transitions with incident photon energy *ħω*_i_, the incident photon excites an electron from the valence band (VB) to the *c*_11_/*c*_10_ state leaving behind a hole. In transitions with scattered photon energy *ħω*_s_, an electron in the *c*_01_/*c*_00_ state recombines with the hole and emits the scattered photon. The incident photon energy *ħω*_i_ and scattered photon energies *ħω*_s_ are linked by energy conservation *E* = *ħω*_i_−*ħω*_s_, where *E* is the energy of AG excitations being studied. In SDE, the electrons are excited from a lower populated AG subband to the next higher subband without the change in AG band index. Red (black) line defines SDE exciting electron states with spin-flip processing from *c*_01_ and *c*_11_ (from *c*_00_ and *c*_10_) under higher (lower) *ħω*_i_. While the *c*_01_ band is above the Fermi energy it is populated because of the thermal excitation of charge carriers at finite temperature of our experiments. **b** Color plot of RILS spectra as a function of *ħω*_i_ measured at temperature *T* = 5 K with crossed polarization. The color bar indicates intensities of scattered light. The dashed lines marked with *c*_01_ → *c*_10_ and *c*_00_ → *c*_11_ indicate the energies of combined intersubband transitions with the change in both quantum well subband and AG band index (see ref. ^25^ and Supplementary Note 2). The dashed line marked with *E*_10_ indicates the position of quantum well subband spacing (see Supplementary Note 2). The yellow dashed lines indicate the position of SDE. There is a weak photoluminescence that overlaps the *c*_01_ → *c*_10_ and *c*_00_ → *c*_11_ transitions, as well as SDE. **c** The dependence of exchange energies extracted from (**b**) on *ħω*_i_. The vertical error bars represent estimated uncertainties in determination of *E*_10_ and *E*_SDE_ from the measured spectra

A large exchange interaction is observed in the spin-density intersubband excitation (SDE) shown in Fig. 4, in which there is no change in the states of the AG potential (intersubband excitations under parallel polarization are presented in Supplementary Figures 7 and 8, and in Supplementary Note 4). The exchange energy associated with SDE can be written as *E*_ex = _$(E_{{\mathrm{10}}}^2 - E_{{\mathrm{SDE}}}^2)/E_{10}$^29^, where *E*_SDE_ is the peak position of SDE and *E*_10_ = 20.0 meV is the QW subband spacing (see Supplementary Note 2, Supplementary Figures 2 and 3). Figure 4a displays RILS processes for intersubband transitions that are from *c*_00_ (*c*_01_) band to *c*_10_ (*c*_11_) band in the second QW subband. As shown in Fig. 4a, *c*_00_ → *c*_10_ transitions have a resonance enhancement at incident photon energies *ħω*_i_ that are lower than the resonance for *c*_01_ → *c*_11_ transitions. Figure 4b shows a color plot of the resonance enhancement for SDE modes at around 20 meV that reveals a remarkable dependence of peak positions on *ħω*_i_. This can be explained by the different resonance enhancements in RILS by excitations associated with different transitions. At lower *ħω*_i_ the SDE spectra come from excitations linked to *c*_00_ → *c*_10_ transitions, whilst at higher *ħω*_i_ the spectra are largely due to excitations from *c*_01_ → *c*_11_ transitions. The changes in the position of SDE peaks as a function of *ħω*_i_ in Fig. 4b are attributed to the different exchange interactions in those two transitions. For a low *ħω*_i_ = 1552.5 meV the SDE mode is at *E*_SDE_ = 19.8 meV and the exchange energy is *E*_ex_ = 0.43 ± 0.05 meV for *c*_00_ → *c*_10_ transitions. The large exchange interaction confirms the results obtained in the saddle-point spin-exciton mode. For a high *ħω*_i_ = 1554.0 meV the SDE mode is at *E*_SDE_ = 19.9 meV and the exchange energy is *E*_ex_ = 0.17 ± 0.05 meV for *c*_01_ → *c*_11_ transitions. The dependence of *E*_ex_ on *ħω*_i_ is shown in Fig. 4c.

The much smaller exchange energy for *c*_01_ → *c*_11_ transitions is attributed to lower carrier population in the *c*_01_ band of AG, since exchange Coulomb energies are proportional to carrier densities. While the *c*_01_ band is above the Fermi energy it is populated because of the thermal excitation of charge carriers at finite temperature of our experiments. Data points in Fig. 4c suggest *E*_ex_ (*c*_00_ → *c*_10_)/*E*_ex_ (*c*_01_ → *c*_11_) ~ 3. According to the Fermi-Dirac distribution at the temperature 5 K, we estimate *n*_*c*00_/*n*_*c*01_ ~ 3, where *n*_*c*00_ and *n*_*c*01_ are carrier densities in the *c*_00_ and *c*_01_ bands, showing *E*_ex_ (*c*_00_ → *c*_10_)/*E*_ex_ (*c*_01_ → *c*_11_) ~  *n*_*c*00_/*n*_*c*01_ ~ 3. Furthermore, as shown in Supplementary Note 8 and in Supplementary Figures 1,1–1,3, sample III has a higher Fermi energy, so that *n*_*c*00_ and *n*_*c*01_ are very close. Then, the determined exchange energies for *c*_01_ → *c*_11_ and *c*_00_ → *c*_10_ transitions, with similar densities, are comparable. The tuning of *E*_ex_ by the electron density provides an effective way to control saddle-point spin-exciton modes and for future studies of excitonic ground states.

Exchange interactions in low-energy excitations (*c*_00_ → *c*_01_) and SDE (*c*_00_ → *c*_10_) are linked to the carrier density in the *c*_00_ band, determining similar exchange energies in these excitations. On the other hand, transitions in *c*_00_ → *c*_01_ and *c*_00_ → *c*_10_ have different values of matrix elements *β*, which would result the difference between the exchange energies (0.6 meV in *c*_00_ → *c*_01_ transitions and 0.45 meV in *c*_00_ → *c*_10_ transitions). At a carrier density of 0.4 × 10^11^ cm^−2^ in the *c*_00_ band, we have *β* ≈ 0.7 × 10^−11^ cm^2^ meV^−1^ for the low-energy excitations based on *c*_00_ → *c*_01_ transitions, and *β* ≈ 0.5 × 10^−11^ cm^2^ meV^−1^ for the SDE based on *c*_00_ → *c*_01_ transitions. It is known that exchange Coulomb interactions in as-grown GaAs QW are strong due to large values of *β*^29^. Values of *β* we obtained in the AG sample are in the same range as the as-grown samples (0.8 × 10^−11^ cm^2^ meV^−1^ measured in ref. ^29^), resulting in large exchange interactions.

## Discussion

The realization of low-disorder AG in small period triangular antidot lattices, which reveals the presence of collective terahertz saddle-point spin excitons at the M-point singularity in the density of states. We observe relatively large exchange Coulomb interactions, which have energies comparable to the gap of Dirac bands at the M-point. Low-disorder AG is thus a highly tunable condensed-matter system for the investigation of graphene-like physics in a regime where the interplay between electron interactions and the hexagonal topology of AG lattices becomes predominant. The large exchange coupling to the honeycomb lattice with tunable parameters makes low-disorder AG lattice potentially suitable for further studies of exciton liquids and spintronics^30^. To realize possible exciton liquids^31,32^, the suppression of Landau damping is required, which would be achieved by opening a gap at K (K’) points (through removing inversion symmetry of the AG lattice). Our findings may open approaches for the investigation of strongly correlated quantum phases in condensed-matter systems such as ferromagnetism and unconventional superconductivity^2,3^, expanding the tool box for quantum simulation. The low-disorder processing approach can be applied to artificial lattices with intriguing topologies such as the Lieb^33,34^ and Kagome lattices. Given that the saddle-point excitons are in the terahertz frequency range and subject to tunable parameters, low-disorder AG may provide a semiconductor platform for optoelectronic device applications.

## Methods

### Fabrication of the AG nanopattern

The semiconductor system we used is 25-nm-wide one-side modulation-doped Al_0.1_Ga_0.9_As/GaAs QW. The QW is positioned 80 nm below the surface and 30 nm below the Si δ-doping layer. The as-grown electron density is 2.1 × 10^11^ cm^−2^, with a Fermi energy *E*_F_ of 7.5 meV and a typical low-temperature mobility of 10^6^ cm^2^ V^−1^ s^−1^. Electron-beam lithography at 80 kV and beam current 400 pA (e-beam writer nB4, NanoBeam Ltd.) was employed to pattern 200 × 200 µm^2^ triangular arrays of circles. We used a single layer resist of Zep 520 as a hard mask, which was developed in a solution of n-Amyl acetate: isopropanol in composition 1:3 with ultrasound sonication. In the development, the temperature is always kept at 0°. After development, a 3 kV electron beam was flood exposed on the pattern to harden the developed resist. An Oxford PlasmaPro System 100 was used to perform the inductively coupled plasma reactive-ion etching. The gas employed was a mixture of 50 sccm Ar and 10 sccm BCl_3_, shown to etch AlGaAs/GaAs heterostructures with high anisotropy with the etching time 60 s. The reactive-ion etching power and ion coupled plasma power were 45 and 200 W with a pressure of 8 mtorr. The residual resist was removed with dimethylacetamide. The etch recipe was optimized to achieve a high degree of physical bombardment and reduced chemical etching.

### Optical measurements

The sample was mounted in an optical cryostat with a base temperature of 4 K. The RILS experiments were performed in a back-scattering configuration, with incident laser beam almost perpendicular (within 10°) to the sample. A tunable Ti:sapphire laser with spot diameter of around 200 µm and power as low as 20 μW was focused on the AG nanopattern. Spectra were collected using a liquid nitrogen cooled CCD through a double grating spectrometer (Spex 1404).

### Data availability

The authors declare that the data that support the plots within this paper and other findings of this study are available from the corresponding author upon reasonable request.

## Electronic supplementary material

Supplementary Information
