# Supplementary material for: Emerging many-body effects in semiconductor artificial graphene with low disorder
Source: Nat Commun. 2018 Aug 17;9:3299. doi: 10.1038/s41467-018-05775-4 (PMC6098128; doi:10.1038/s41467-018-05775-4)
Supplement: Supplementary file 1 — Supplementary Information [file 41467_2018_5775_MOESM1_ESM.pdf]

Emerging many-body effects in semiconductor artificial graphene with low disorder

Du et al

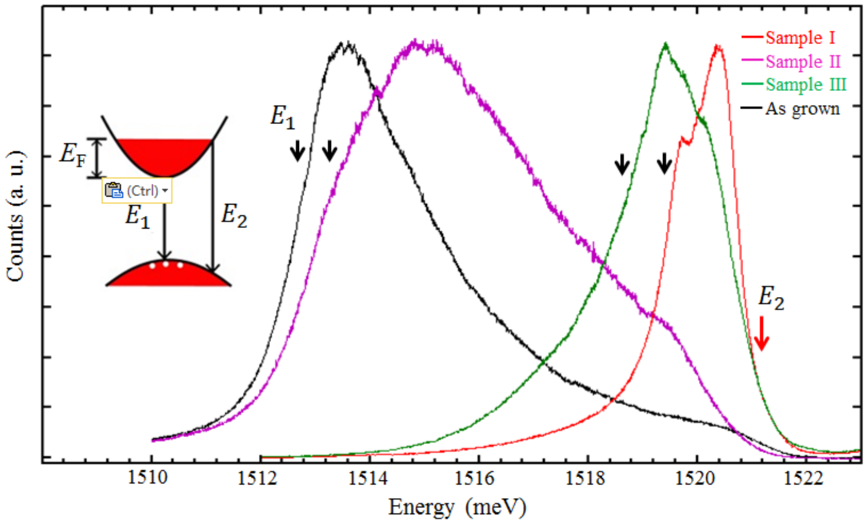

Supplementary Figure 1. Photoluminescence spectra of different samples. The black (red) arrows mark the positions of E_1 (E_2). E_1 and E_2 represent the energies of transitions at the bandgap and the Fermi energy as displayed in the inset.

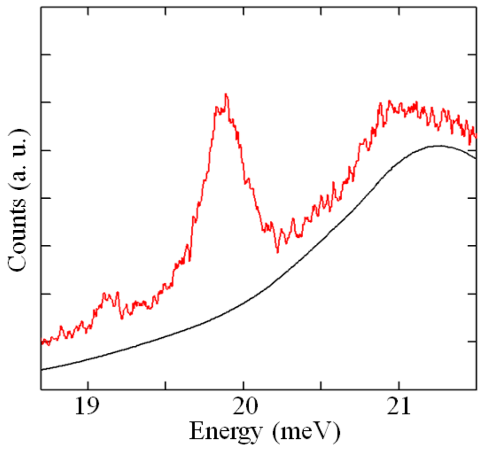

Supplementary Figure 2. Example of background subtraction in resonant inelastic light scattering spectra. The red line is the resonant inelastic light scattering spectrum measured at incident laser photon energy $\hbar\omega_{\mathrm{i}}$ = 1554.7 meV. The black line is the photoluminescence signal measured at larger photon energy. The subtracted trace is shown in Supplementary Figure 3.

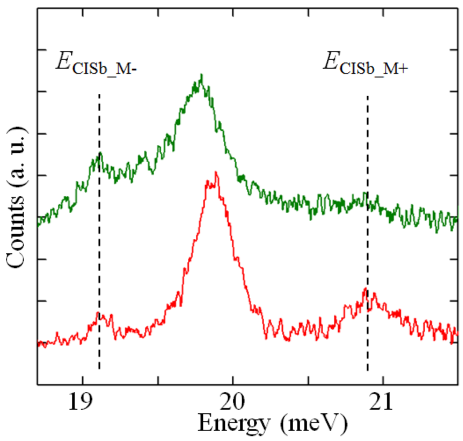

Supplementary Figure 3. Resonant inelastic light scattering spectra of combined intersubband transitions. Spectra are measured at $\hbar\omega_i$ = 1554.7 meV (red) and 1552.1 meV (green)**.** The dashed line near 19.1 meV is for $c_{01} \rightarrow c_{10}$ transitions near the M-point (E_{CISb_M-}) and the dashed line near 20.9 meV is for $c_{00} \rightarrow c_{11}$ transitions near the M-point (E_{CISb_M+}).

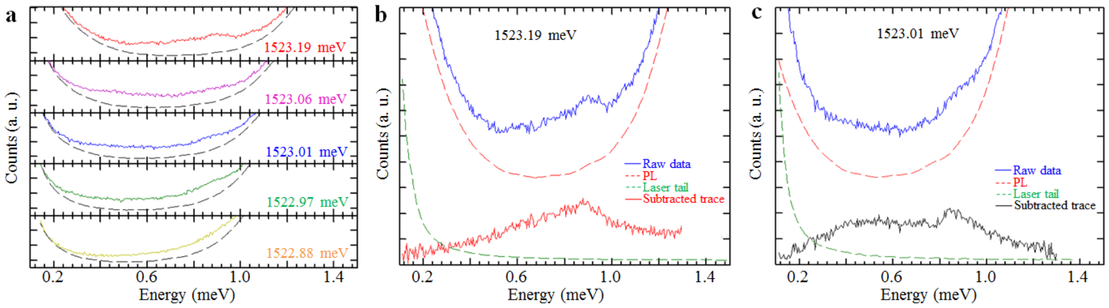

Supplementary Figure 4. Subtraction of background in resonant inelastic light scattering (RILS) spectra of low-energy excitations. (a) shows cross-polarized resonant inelastic light scattering spectra (solid traces) of low-energy Dirac band transitions from sample I. The incident photon energies are indicated. The dashed lines are background intensities that include photoluminescence (PL) and the laser tail. (b) and (c) show the subtraction in two RILS spectra. The incident photon energies are indicated. The blue lines are the raw data. The red dashed lines are PL measured at larger exciting photon energy (1526.34 meV) and the green dashed lines are the laser tail measured at larger photon energy (1527.14 meV). The red solid line in (b) and the black sold line in (c) are the resonant inelastic light scattering traces after subtraction. The laser tail and subtracted traces are shifted vertically for clarity.

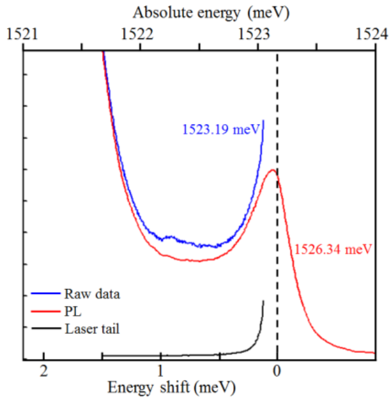

Supplementary Figure 5. Plot of the resonant inelastic light scattering spectrum and the photoluminescence (PL) trace in Supplementary Figure 4(b) in the absolute energy scale and the relative energy scale. Exciting photon energies of the spectrum and PL trace are noted in colors of blue and red, respectively. The laser tail is also plotted in the relative energy scale. The dashed line indicates the position of the exciting photon energy for the resonant inelastic light scattering spectrum.

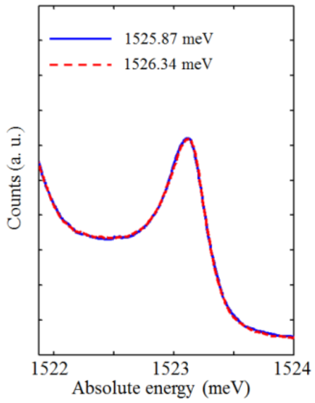

Supplementary Figure 6. Photoluminescence traces for different exciting photon energies in the absolute energy scale.

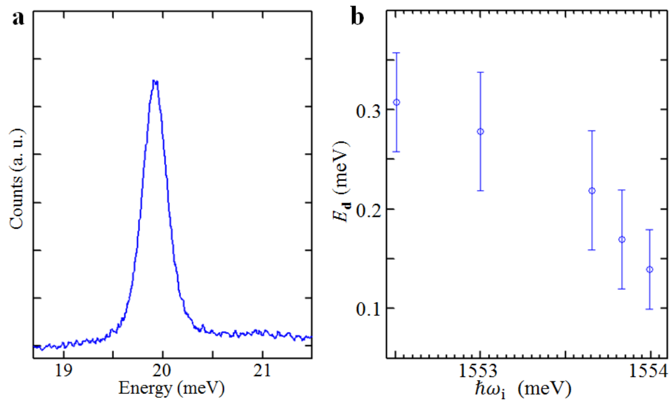

Supplementary Figure 7. Intersubband charge density excitations. (a) Resonant inelastic light scattering spectrum of intersubband charge density excitation at $\hbar\omega_\mathrm{i}$ = 1553 meV. (b) Direct Coulomb interaction term we obtained from intersubband charge density excitations. The error bars represent estimated uncertainties in determinations of E_SDE and E_CDE from the measured spectra

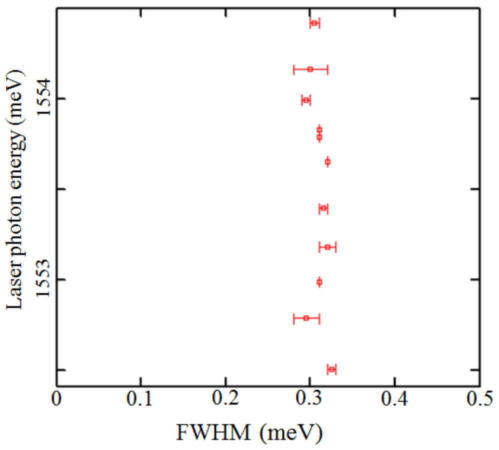

Supplementary Figure 8. Full width at half maximum (FWHM) of intersubband spin density excitation peaks in sample I. The error bars represent estimated uncertainties in determination of FWHM from the measured spectra.

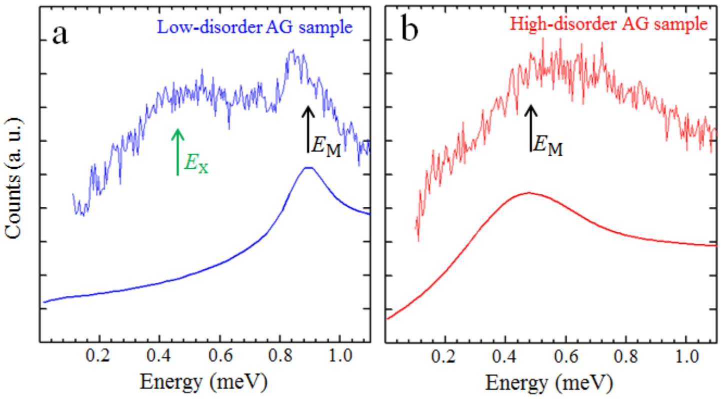

Supplementary Figure 9. Comparison of the low-energy resonant inelastic light scattering (RILS) spectrum in low-disorder artificial graphene with that in a control sample in an artificial graphene honeycomb dot lattice with much higher disorder in Supplementary Reference 1. Upper panel in (a) shows the RILS spectrum in low-disorder artificial graphene. Black arrows show the calculated positions of the M-point singularity. A green arrow indicates the E_X mode. Lower panel in (a) shows joint density of states (JDOS) calculated for transitions between c_{00} and c_{01} states with the parameters of sample I in Supplementary Table 1. The JDOS is broadened by Gaussian disorder of width 0.1 meV. Upper panel in (b) shows the RILS spectrum in the artificial graphene with higher disorder. Lower panel in (b) shows the JDOS calculated for transitions between c_{00} and c_{01} states with a = 50 nm, dot radius r = 8.5 nm, artificial graphene potential V = -6.9 meV and E_F = 0.9 meV. The JDOS is broadened by Gaussian disorder of width 0.3 meV.

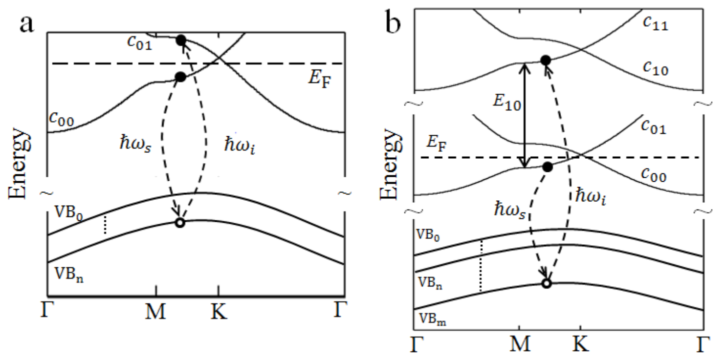

Supplementary Figure 10. Optical transitions in resonant inelastic light scattering (RILS). (a) Optical transitions in RILS of low energy spin-density excitations are indicated. To simplify discussions, parabolic bands are used to depict valence subbands. Band diagrams are not scaled. In transitions with incident photon energy $\hbar\omega_i$, the incident photon excites an electron from a valence subband VB_n to the c_{01} state leaving behind a hole. In transitions with scattered photon energy $\hbar\omega_s$, an electron in the c_{00} state recombines with the hole and emits the scattered photon. The incident photon energy $\hbar\omega_i$ and scattered photon energies $\hbar\omega_s$ are linked the by energy conservation $E = \hbar\omega_i - \hbar\omega_s$, where E is the energy of excitations being studied. (b) Optical transitions in RILS of intersubband excitations are indicated. E_{10} indicates quantum well subband spacing in the conduction band. In transitions with $\hbar\omega_i$, the incident photon excites an electron from a valence subband VB_m to the c_{10} state leaving behind a hole. In transitions with $\hbar\omega_s$, an electron in the c_{00} state recombines with the hole and emits the scattered photon.

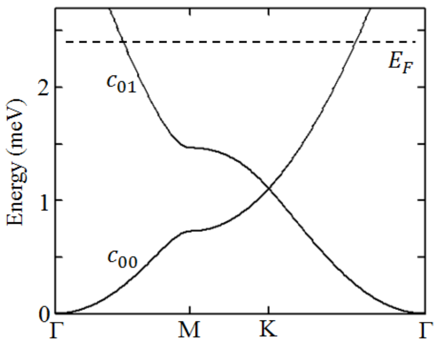

Supplementary Figure 11. The two lowest Dirac bands of artificial graphene sample III with the parameters in Supplementary Table 1.

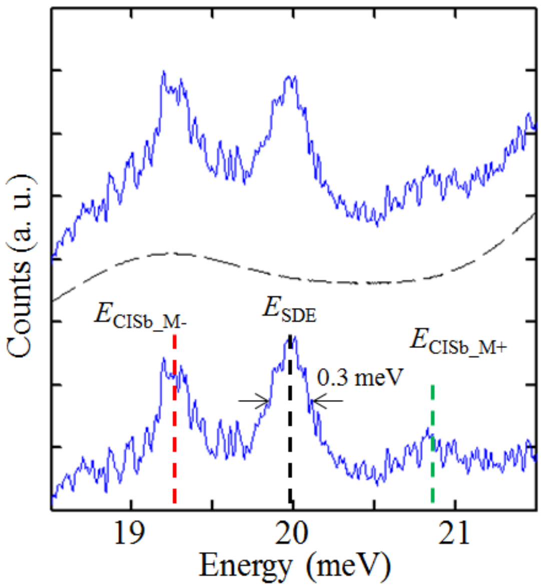

Supplementary Figure 12. Resonant inelastic light scattering spectrum of intersubband excitations and background subtraction in sample III. The spectrum in the upper panel is measured at $\hbar\omega_{\mathrm{i}}$ = 1552.8 meV**.** The black dash line is the photoluminescence signal measured at larger photon energy. The lower panel shows the subtracted trace. Similar with those in Supplementary Figure 2, the dashed red line near 19.3 meV is for $c_{01} \rightarrow c_{10}$ transitions near the M-point ($E_{\mathrm{CISb\_M-}}$) and the dashed green line near 20.87 meV is for $c_{00} \rightarrow c_{11}$ transitions near the M-point ($E_{\mathrm{CISb\_M+}}$). The black dashed line marks the position of intersubband spin density excitations (E_{SDE}). The marked width of intersubband spin density excitation is 0.3 meV.

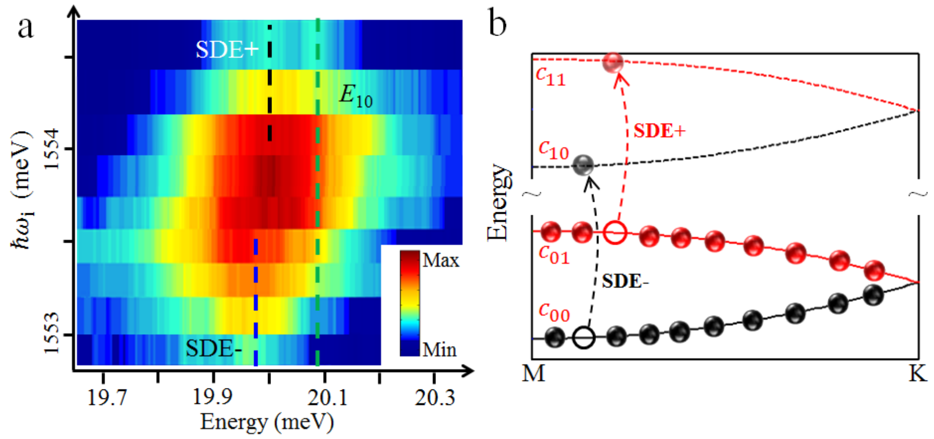

Supplementary Figure 13. Intersubband spin density excitations in sample III. (a) Color plot of intersubband spin density excitation spectra for incident photon energy $\hbar\omega_i$ in sample III. SDE+ and SDE- denote two regimes of spin density excitations with different exchange Coulomb terms. (b) Transitions in resonant inelastic light scattering of intersubband spin-density excitations are indicated. Red (black) line defines intersubband spin-density excitations exciting electron states from c_{01} and c_{11} (from c_{00} and c_{10}) with higher (lower) $\hbar\omega_i$. The color bar indicates intensities of scattered light.

Supplementary Table 1. Summary of parameters of artificial graphene samples.

sample	period *b* (nm)	etching depth *d* (nm)	hole radius *r* (nm)	lattice constant *a* (nm)	Fermi energy E_F (meV)
I	70	48	20	40.4	1.4
II	60	25	12	34.6	7.2
III	80	48	22	46.1	2.4

Supplementary Note 1: Determination of the Fermi Energy and the potential of the AG patterns

The Fermi energy E_F is determined from photoluminescence (PL) spectra of optical emission for transitions between conduction and valence subbands.

In Supplementary Figure 1, the Fermi energy can be calculated with the parabolic dispersion approximation:

$$E_F = \frac{m_h}{m_e + m_h}(E_2 - E_1) \ , \qquad (1)$$

where m_e and m_h are effective masses for conduction and valence bands, respectively. The uncertainty in the Fermi energy determination is ~ 0.1 meV. The Fermi energy of the as-grown quantum well is 7.8 meV and the Fermi energies of other samples are shown in Supplementary Table 1.

In the nano-fabricated devices, the binding energy of donors in GaAs is about 5 meV[1,2]. In sample I and III, PL spectra measured outside the pattern reveal full depletion of the electron density, suggesting that the confinement potential V_0 should be larger than 5 meV. Considering that the Fermi energy is about 1-2 meV, we employ $V_0 = 6$ meV. Due to a small etching depth, sample II is weakly modulated by the potential with AG band structure not fully developed. It is suggested by the PL spectrum of sample II which is similar with that of as-grown quantum well.

Supplementary Note 2: Combined intersubband transition: determination of the single particle intersubband energy in sample I

A weak PL signal superimposed on resonant inelastic light scattering (RILS) spectra of intersubband excitations is subtracted from the raw data to reveal the RILS transitions. The details of the subtraction are illustrated in Supplementary Figure 2. In Supplementary Figure 3, $E_{\mathrm{CISb\_M+}}$ and $E_{\mathrm{CISb\_M-}}$, are energies of combined intersubband excitations in $c_{00} \rightarrow c_{11}$ and $c_{01} \rightarrow c_{10}$ transitions[2] near the M point. Thus the QW subband spacing E_{10} satisfies: $E_{10} = E_{\mathrm{CISb\_M-}} + E_{\mathrm{M}}$ and $E_{10} = E_{\mathrm{CISb\_M+}} - E_{\mathrm{M}}$. The QW subband spacing E_{10} could be obtained from $E_{10} = (E_{\mathrm{CISb\_M-}} + E_{\mathrm{CISb\_M-}})/2 = 20.0$ meV.

Supplementary Note 3: Background subtraction in resonant inelastic light scattering spectra of low-energy Dirac band transitions in sample I

Since here RILS occurs at low energies (~ 0.5 meV), a slopping background due to the laser tail overlaps the RILS signal[1]. Moreover, we find a weak PL signal that overlaps the RILS spectra. This PL signal originates in optical transitions from the c_{00} states to higher energy (above the ground state) holes in valence subbands[1]. The optical transitions overlap the conduction to valence transitions that resonantly enhance RILS. Backgrounds that originate in the laser tail and the weak PL are subtracted from the raw data to reveal the RILS spectra of low-energy Dirac band transitions, as shown in Supplementary Figure 4. Supplementary Figure 4(a) shows the measured intensity (solid lines) and the background signals (dashed line), and Supplementary Figures 4(b) and 4(c) show the detailed subtraction procedure. The background signals in Supplementary Figure 4(a) are the combination of the PL signal and the laser tail. The PL signal we used in Supplementary Figure 4(a) is the same for different exciting photon energies in the absolute energy scale. Supplementary Figure 5 shows a single spectrum and the PL signal in the absolute and relative energy scales. At absolute energies below the RILS feature, the spectrum coincides with the PL trace. Moreover Supplementary Figure 6 shows that the PL traces are nearly the same in the absolute energy scale for larger exciting photon energies where RILS features do not emerge.

Supplementary Note 4: Intersubband charge density excitations under parallel polarization in sample I

Under parallel polarization, intersubband charge density excitations (CDE) can be measured by RILS, as shown in Supplementary Figure 7(a). The peak of CDE has a large intensity that obscures signals of combined intersubband excitations. The energies of charge density modes are shifted from the energies of spin density modes due to direct Coulomb interactions[3]. As shown in Supplementary Figure 7(b), we obtain the direct Coulomb interaction term[3] $E_{\mathrm{d}} = (E_{\mathrm{CDE}}^2 - E_{\mathrm{SDE}}^2)/E_{10}$, where E_{CDE} is the peak position of CDE, E_{SDE} is the peak position of SDE and E_{10} = 20.0 meV is the QW subband spacing. The direct Coulomb interaction terms we obtained are comparable to the exchange energies shown in Fig. 4c.

Supplementary Note 5: Widths of intersubband spin density excitation peaks in sample I

Supplementary Figure 8 shows that the widths of intersubband spin density excitation peaks are not dependent on laser photon energies.

Supplementary Note 6: Alternative interpretation for the peak E_X in low energy excitations.

It is conceivable that the peak E_X is from defects created by nano-patterning. We can exclude this possibility from the following reasons.

The E_X peak is not seen in samples with higher disorder. To show this, we compare in Supplementary Figure 9 the RILS spectrum in the low-disorder sample (AG sample I) with the one from a "control sample" in Supplementary Reference 1, which is from a 50nm period AG lattice with much higher disorder. Supplementary Figure 9 shows that the higher disorder results a single broader M-point peak but does not bring any additional peaks below the M-point peak. This shows that the main impact of defects is to broaden the features in low energy excitations, so that the peak E_X in sample I cannot be interpreted by defects.

If the E_X peak is a defect-induced mode, the absence of the spin density excitation peak in low energy spectra would suggest negligible exchange interactions, which is inconsistent with calculations of exchange energies at the carrier densities of our sample.

If the E_X peak is a defect-induced mode, the mode would contribute to intersubband excitations and we should expect three peaks in intersubband excitations. The first one is a spin density excitation peak with energy at the quantum well subband spacing due to negligible exchange interactions. The second one is from the upper gapped state in the c_{01} band to the lower gapped state in the c_{10} band, with about 0.45 meV below the first one. The third one is from the lower gapped state in the c_{00} band to the upper gapped state in the c_{11} band, with about 0.45 meV above the first one. Apparently, these features are not consistent with intersubband spectra shown in Fig. 4b.

SDE provides additional measurement to the exchange energy. In low energy excitations, electrons are excited from the c_{00} band to the c_{01} band with the exchange interaction between electrons in the c_{00} band and excited electrons in the c_{01} band. In SDE (c_{00} to c_{10}), electrons are excited from the c_{00} band to the c_{10} band with the exchange interaction between electrons in the c_{00} band and excited electrons in the c_{10} band. Exchange Coulomb energies are proportional to carrier densities. Thus the electron density in the c_{00} band is linked to the exchange energies in both the low energy excitations and the SDE. In the SDE, we obtain the exchange energy of about 0.5 meV. Then in the low energy excitations, we could expect that a spin density excitation peak is red-shifted from the M-point singularity by a similar exchange energy and is around 0.5 meV. Since we observe only one additional mode around this energy, it is reasonable to attribute it to the spin density excitation due to the exchange interaction.

Supplementary Note 7: Optical transitions in RILS in sample I

Optical transitions in RILS of low energy spin-density excitations are plotted in Supplementary Figure 10(a). Supplementary Figure 10(a) shows that the optical transitions in our RILS experiments are from a higher valence subband state VB_n. In this fashion, the RILS signals do not overlap the strong main PL associated to E_2. On the other hand, optical transitions in RILS of intersubband excitations are plotted in Supplementary Figure 10(b). Supplementary Figure 10(b) shows that the optical transitions in intersubband excitations are from a higher valence subband state VB_m. Since RILS signals in intersubband excitations are weak, the valence subband VB_m is higher than VB_n so that the RILS signals do not overlap strong PL signals. The valence subband VB_m supports a weak PL with energy around 1533.5 meV, which is shown in Fig. 4b and Supplementary Figure 2.

Supplementary Note 8: Intersubband spin density excitations in sample III

The Fermi energy in sample III is 2.4 meV. As shown in Supplementary Figure 11, both c_{00} and c_{01} bands near the M point are populated, and thus the low energy mode transition ($c_{00} \rightarrow c_{01}$) is forbidden due to the Pauli exclusion principle. Supplementary Figure 12 shows three RILS peaks. The SDE peak shows a narrow full width at half maximum of 0.3 meV, which is comparable with that of sample I. The sharp SDE peak here confirms low disorder in the antidot AG devices. The RILS peaks (E_{CISb_M+} and E_{CISb_M-}) are from the large JDOS for $c_{00} \rightarrow c_{11}$ and $c_{01} \rightarrow c_{10}$ transitions near the M point. With similar steps we described in Supplementary Note 2, we have E_{10} = 20.09 meV for sample III.

As shown in Supplementary Figure 13(a), the SDE peaks are red-shifted from E_{10}, which is linked to exchange Coulomb interactions. Due to the higher Fermi energy in sample III, both c_{00} and c_{01} bands near the M point are populated. In this configuration, the electron densities at the M points of c_{00} and c_{01} bands are very close, allowing us to probe the density dependence of exchange energies. At lower $\hbar\omega_i$ the SDE spectra come from excitations linked to $c_{00} \rightarrow c_{10}$ transitions (SDE-), whilst at higher $\hbar\omega_i$ the spectra are largely due to excitations from $c_{01} \rightarrow c_{11}$ transitions (SDE+), as shown in Supplementary Figure 13(b). In Supplementary Figure 13(a) energies of intersubband peaks for SDE+ and SDE- are very close, showing that the difference between exchange energies for $c_{00} \rightarrow c_{10}$ and $c_{01} \rightarrow c_{11}$ transitions is small. For low $\hbar\omega_i$ = 1553 meV, the SDE mode is at E_{SDE} = 19.98 meV and the exchange energy is E_{ex} = 0.22 meV for $c_{00} \rightarrow c_{10}$ transitions. For high $\hbar\omega_i$ = 1554.5 meV, the SDE mode is at E_{SDE} = 20 meV and the exchange energy is E_{ex} = 0.18 meV for $c_{01} \rightarrow c_{11}$ transitions. The difference between these two values is much smaller than that in sample I as shown in Fig. 4c.

Supplementary References

1. Wang, S. *et al.* Observation of Dirac Bands in Artificial Graphene in Small Period Nano-patterned GaAs Quantum Wells. *Nature Nanotech.* **13**, 29-33 (2017).
2. Wang, S. et al. Observation of electron states of small period artificial graphene in nano-patterned GaAs quantum wells. *Appl. Phys. Lett.* 109, 113101 (2016).
3. Pinczuk, A. *et al*. Large Exchange Interactions in the Electron Gas of GaAs Quantum Wells. *Phys. Rev. Lett.* **63**, 1633-1636 (1989).
